# Dr. Paine's Local Anesthetic

**Published:** 1896-01

**Authors:** 


ARTICLE II.'

### DR. PAINE'S LOCAL ANESTHETIC.

President Younger.—In reference to Dr. Payne's anesthetic, give your opinion of its actions, contrasts and merits. As for my anesthetic, have no delicacy on my account. If as good as it appears I shall certainly abandon mine for it.

Dr. Merriman, Jr.—The immediate effect of the new anesthetic was astonishing. After injecting an anesthetic we usually wait a few moments, but with this the Doctor was able to act at once. The patients apparently felt no pain, particularly the young lady, whom I watched very closely.

Dr. Geo. W. Cool.—I fully appreciate the work of Dr. Payne's anesthetic from the experience I have had in the use of local anesthetics; I don't know of one that localizes the anesthetic effect so rapidly. I was particularly struck with Dr. Younger's anesthetic, but, although a success in his hands, I doubted its success in mine.

Dr. Cecil Corwin.—I had a personal experience with Dr. Payne's anesthetic in my own mouth as he operated on my gums. The anesthetic was the most immediate and profound of any I have ever used in my mouth. Have also had Dr. Wilson's and Dr. Younger's applied to my gums. They have their merits. Wilson's is good in some cases; Younger's a little better, but I think we have now something still better. This clinic alone has repaid me for all my time in attending the meetings of the Stomatological Club.

Dr. R. H. Cool.—I was thinking of the use of nitro-glycerine in this compound. A year ago I advised the use of nitro-glycerine with cocaine. I still insist that such should be the case. I notice that the preparation put on the market by S. S. White & Co. for Dr. Kirk contains nitro-glycerine. I think every anesthetic made hereafter will contain it; no matter how small the amount used, it will have a stimulating effect. I always use a cardiac stimulant with the application of cocaine. Dr. Payne in his formula insists on the use of chemically pure German glycerine. He says that the ordinary glycerine contains arsenic, which probably accounts for the sloughing when using it. The effect to-day was so pleasing to me that I wish to thank Dr. Payne personally, and I shall certainly use his anesthetic.

Dr. F. C. Pague.—One point in the clinic caught my eye, and I took it home to myself; I fell on it by accident. In the introduction of the hypodermic syringe I have been in the habit of finding the soft tissue. I accidentally took a needle and run it into the hard tissue flat, as Dr. Payne demonstrated to-day, and the patient did not move. On the opposite side I run into the soft tissue with contrary results. Hunt the hard spots and introduce the point flat.

Dr. Cummings.—The Doctor's manner of introducing the needle, I accidentally found out, makes a good deal of difference. Press the tissue to keep the blood away. The anesthetic worked very nicely, and I am very thankful to Dr. Payne for the formula.

Dr. Younger.—The needle was very short and could hardly be introduced over half an inch. How could it reach apex of root to obtund the nervous texture passing out of the apex, unless we accept the statement of Dr. Brophy and Black, "that the inferior dental nerve gives no filament to the dental pulp, and the sensation is due to lateral means," In using my anesthetic I have tried to get as near those parts as possible and obtund to prevent pain. Dr, Payne did not insert even half of an inch, and extraction was accomplished without any pain. It is the best anesthetic I have ever seen, and is away ahead of mine. I am very glad to find something better and I feel under obligations to Dr. Payne; I shall certainly abandon mine until I find it is better. He did not go into the soft tissues at all, yet the insensibility produced in that half of an inch was enough to obtund the sensibility in the whole tooth, and that is what surprised me to day.

A member.—Q. I would ask Dr. Payne if he has ever had any ill effects? A. None at all.

Dr. Lundborg.—I observed that in injecting the anesthetic the Doctor did not seem to benumb the gum with the anesthetic before inserting the needle. Was not there some sensation? I have found in injecting cocaine that by putting the needle in boiling water, then injecting hot water, and after that inject the anesthethic, that it works splendid; there is no sensation.

Dr. Greenlaw.—I have been using a local anesthesia for sometime. By dipping the needle in carbolic acid first it relieves pain.

Dr. Clyde Payne.—I am pleased beyond expression at hearing so much in favor of my clinic. I will endeavor to explain what caused me to select the formula. Cocaine is the base of nearly all local anesthetics. There are a great many forms of cocaine. Cocaine being worth $5 an ounce makes it worth counterfeiting; hence, there are a great many counterfeits on the market. I have used all the different brands from the reputable houses, but from none do I get results the same as from Merck's. In regard to the solution, I used 10 per cent. and got some ill physiological effects. I stopped it and tried 5 per cent., with fair results. In one particular case the patient turned pale and showed the ill-effect of cocaine. I came to the conclusion, as it depressed the heart, that there must be some excitant. I looked around for a heart stimulant. If you use digitalis it must be made fresh. When it comes in contact with the blood it loses its alkalinity. Nitro-glycerine is not affected by the blood. It stimulates the heart and counteracts the effect of cocaine. Cocaine contracts the capillary blood-vessels; nitro-glycerine dilates them. If you use ordinary glycerine the overheating in its production makes it irritating instead of soothing. There is only one chemically pure glycerine on the market—a German double-distilled. When you force glycerine into any part it anesthetizes that part and localizes it. In inserting the needle, press the part, have needle flat and keep the anesthetic ahead of the needle. After inserting as far as you can, you use force on the piston, and you not only get to the apex but you also get infiltration. In anesthetizing part at the end of the root, you push flat side down in between tooth and root, and if the pericementum is in good condition it will act all the better. When conditions are healthy there is quite a space in this cushion, and you can force the anesthetic to the apex of the root. You must keep away from pus socks if there is an abscess. If you get in, the sack being full, it overflows, gets into the tissue and makes trouble. Keep out of the soft tissue; keep in the mucous gum.—*Proceedings of Stomatological Club.*

